# Concentration: The Neural Underpinnings of How Cognitive Load Shields Against Distraction

**DOI:** 10.3389/fnhum.2016.00221

**Published:** 2016-05-18

**Authors:** Patrik Sörqvist, Örjan Dahlström, Thomas Karlsson, Jerker Rönnberg

**Affiliations:** ^1^Department of Building, Energy and Environmental Engineering, University of GävleGävle, Sweden; ^2^Linnaeus Centre HEAD, Swedish Institute for Disability Research, Linköping UniversityLinköping, Sweden; ^3^Department of Behavioral Sciences and Learning, Linköping UniversityLinköping, Sweden; ^4^Center for Medical Image Science and Visualization (CMIV), Linköping UniversityLinköping, Sweden

**Keywords:** working memory, selective attention, concentration, cognitive load, distraction

## Abstract

Whether cognitive load—and other aspects of task difficulty—increases or decreases distractibility is subject of much debate in contemporary psychology. One camp argues that cognitive load usurps executive resources, which otherwise could be used for attentional control, and therefore cognitive load increases distraction. The other camp argues that cognitive load demands high levels of concentration (focal-task engagement), which suppresses peripheral processing and therefore decreases distraction. In this article, we employed an functional magnetic resonance imaging (fMRI) protocol to explore whether higher cognitive load in a visually-presented task suppresses task-irrelevant auditory processing in cortical and subcortical areas. The results show that selectively attending to an auditory stimulus facilitates its neural processing in the auditory cortex, and switching the locus-of-attention to the visual modality decreases the neural response in the auditory cortex. When the cognitive load of the task presented in the visual modality increases, the neural response to the auditory stimulus is further suppressed, along with increased activity in networks related to effortful attention. Taken together, the results suggest that higher cognitive load decreases peripheral processing of task-irrelevant information—which decreases distractibility—as a side effect of the increased activity in a focused-attention network.

## Introduction

When people are mentally engaged in a challenging or entertaining task, they sometimes fail to notice what is going on in the surrounding environment. Whilst driving alone on a strait highway, for example, we easily pick up what is said on the radio in the background, but as we cross the center of a large city in heavy traffic, and maneuvering becomes more complex, we may even fail to notice our children calling from the back seat. Situations such as this, when higher task difficulty makes us less likely to detect what is happening in the background, are sometimes referred to as effects of concentration. Here, the concept “concentration” refers to the deliberate attempt to compensate for high task difficulty. As people concentrate, they engage more into the task with the purpose to maintain a desirable level of performance. One consequence of this task-engagement is that concentration reduces peripheral processing and shields against distraction (Sörqvist and Marsh, 2015), which could be why we sometimes fail to notice when someone calls out our own name (Conway et al., 2001).

Our view of mental concentration is that it refers to the ability to selectively attend to a target stimulus and ignore other sources of information (selective attention), and it also refers to the dynamic mechanism of task-engagement. For example, a person at a cocktail party can selectively move the locus-of-attention from talker to talker in the room (selective attention), while either being fully engaged in the listening activity (as when the talker says something interesting or challenging) or not especially engaged (as when the talker is uninteresting). The need for concentration is largely determined by the difficulty of the current task, but task-engagement can also vary with other factors such as motivation and expertise (Sörqvist and Marsh, 2015).

Task demands can vary as a function of perceptual load (i.e., the number of potentially relevant but ultimately irrelevant/non-target sources of information in the current environment), sensory load (i.e., the difficulty with which target information can be perceived) and cognitive load (i.e., the burden posted by the task requirements in relation to the cognitive system’s capacity). For instance, when looking across a town center searching for a specific person, the number of other people present will determine the degree of perceptual load (Jenkins et al., 2005); background noise masking a speech signal determines sensory load in the listening task (Stenfelt and Rönnberg, 2009); and the number of items that have to be kept in mind when solving mental arithmetic determines the task’s cognitive load (Klinger et al., 2011). The conceptual distinction between these forms of load is not always clear cut and their effects on selective attention are debated (Benoni and Tsal, 2013). Although there are reports on the failure to find effects of perceptual load—in the visual modality—on auditory distractor processing (Murphy et al., 2013) and on visual distractor processing (Yeshurun and Marciano, 2013), the vast majority of evidence agrees that perceptual load and sensory load decrease distractor processing (Lavie, 2010; Hughes et al., 2013; Marsh et al., 2015). Furthermore, visual-perceptual load suppresses not only processing of distractors presented in the visual modality (Lavie et al., 2004; Jacoby et al., 2012; Mevorach et al., 2014) but also processing of distractors presented in the auditory modality (Macdonald and Lavie, 2011; Halin et al., 2014a). Moreover, perceptual/sensory load inhibits processing of both externally generated distractors (Halin et al., 2014b)—such as background noise—and internally generated distractors (Forster and Lavie, 2009)—such as task-unrelated thought. Hence, when task difficulty is high—due to perceptual or sensory load—the compensatory upward shift in concentration shields against distractor processing (Linnell and Caparos, 2013). The reasons for this could be that high task difficulty locks the locus of attention to the target information (whereby surprising, rare or unexpected information loses its ability to capture attention) and suppresses the neural processing of task-irrelevant information (Sörqvist and Marsh, 2015).

Whether high cognitive load also shields against distraction, similar to perceptual/sensory load, is more debatable. One source of evidence suggests that cognitive load increases distractor processing (Lavie and De Fockert, 2005; Dalton et al., 2009). On this view, working memory and executive resources are needed to combat distraction, and when cognitive load is high (e.g., when several items have to be maintained in working memory), the necessary resources are usurped, whereby susceptibility to distraction increases (Lavie, 2005). Another source of evidence indicates that higher cognitive load decreases distractor processing (Berti and Schröger, 2003; Kim et al., 2005; SanMiguel et al., 2008; Halin et al., 2015). On this view, cognitive load—just as perceptual and sensory load—increases task difficulty, which reduces susceptibility to distraction, because of the increase in focal task-engagement (i.e., concentration), emerging through the compensatory processes needed to maintain a high level of performance when task difficulty is high (Sörqvist and Marsh, 2015).

As in the context of behavioral data, there are some neuroimaging studies which suggest that higher cognitive load in the attended task (e.g., listening to a speech stream presented in one ear) increases the neural activity in response to the ignored stimuli (e.g., sound presented in the other ear; Sabri et al., 2014); and, conversely, there are some neuroimaging studies which suggest that higher task difficulty in the attended task suppresses the cortical activity in response to distractors. For example, increasing auditory working memory load decreases activity in brain areas serving visual processing (Klemen et al., 2010) and* vice versa* (Zhang et al., 2006; Regenbogen et al., 2012).

These inconsistent effects of cognitive load motivated an empirical replication of the key results from previous studies on the effects cognitive load on auditory processing (Sörqvist et al., 2012). In the experiment reported here, we used an functional magnetic resonance imaging (fMRI) protocol to explore whether cognitive load—as manipulated with a visual-verbal working memory task—influences the cortical processing of background sound. Participants were requested to view a sequence of visual items and indicate whether the current item was the same as the item presented *n* steps back in the sequence. *n* was 1 in the low demand condition and 3 in the high demand condition. A background sound was presented concurrently with the visual items. Participants were told to ignore the sound, except in an active listening condition without a visual task. We hypothesized that active attention to the auditory stimulus would facilitate its processing in auditory cortex, and when the locus of attention is directed towards a visual stimulus instead, the neural response to the auditory stimulus should be attenuated. The auditory cortex’ response to the auditory stimulus should be further suppressed when the difficulty of the visual-verbal working memory task increases. Moreover, the activity in frontoparietal areas and areas involved in monitoring of saliency and effort (i.e., the anterior cingulate and insula) should increase as task difficulty increases (because the burden on working memory is higher; Owen et al., 2005; see also Menon and Uddin, 2010), even though the actual sensory input is kept constant when task difficulty varies.

While the effects of cognitive load on a cortical level have been extensively studied, effects of cognitive load on a subcortical level are—to the best of our knowledge—less explored. The small body of evidence indicates that similar suppression effects of high cognitive load also arise at subcortical stages in the stimulus-processing chain. Selectively attending a visual stimulus reduces brainstem responses to task-irrelevant background sound (Ikeda, 2015). Attending the sound increases the amplitude of the brainstem response; the brainstem response decreases when attention is shifted from the auditory modality toward the visual modality; and the brainstem response is further suppressed when the cognitive load of the visual task increases—even when the visual stimulus input remains the same (Sörqvist et al., 2012).

Because of the theoretical interest in exploring how far the effects of cognitive load reach, our intention here was to expand the analysis of the effects of cognitive load to include a classical and much researched subcortical area—the amygdala (Pessoa et al., 2005; Diekhof et al., 2011; Okon-Singer et al., 2014). The amygdala is known for its role in emotional behavior and receives direct and indirect connections from the posterior thalamus (Doron and Ledoux, 2000). However, the amygdala is also interconnected with the sensory and associative auditory cortices (Bzdok et al., 2013) and hence is involved in sound processing; in particular in the analysis of non-linguistic, environmental sounds (Strobel et al., 2015). For example, hearing the sound of instruments used in dental treatment—a sound that is associated with what many people view as unpleasant—activates the amygdala along with the prefrontal cortex (Yu et al., 2015). Moreover, cognitive load appears to have an effect on amygdala responses (Kellermann et al., 2012; see also Cohen et al., 2015, for pupillary responses). The introduction of a cognitively demanding task can down-regulate the brain’s responses to the negative stimuli in the amygdala, at least when the cognitive task is introduced after exposure to emotionally negative stimuli (Van Dillen et al., 2009). It is unclear, however, whether high cognitive load has corresponding suppression effects on the amygdala’s responsiveness to a stimulus that is not emotionally loaded. An adaptive cognitive system, designed to facilitate selective attention, which deals with a cognitively demanding task might downregulate the activity in amygdala as a preventive measure to shield from the potential influence of emotional responses, even when the environmental stimulus is not emotionally loaded. If this is the case, higher cognitive load in a visual-verbal working memory task might not only suppress the auditory cortex’ responses to a non-emotional task-irrelevant sound, but it might also suppress the amygdala’s responsiveness to the background sound.

To summarize, we were particularly interested in the effects of visual-verbal cognitive load with respect to the activity in the auditory cortex and the amygdala. For the auditory cortex, we used the nomenclature established by Morosan et al. (2001), which is considered more accurate than the conventional notion of an auditory cortex (e.g., Morosan et al., 2005). This categorization divides the auditory cortex into three regions: Te1.0, Te1.1, and Te1.2. In addition, we included the auditory parainsular cortex, Brodmann area (BA) 22. BA2, which is located in the lateral postcentral gyrus and is a prominent part of the somatosensory cortex, was used as a reference, since we did not expect to find effects of sound or effort in this sensory region.

## Materials and Methods

### Participants

A total of 32 students (18 females and 14 males, mean age = 24.97 years) were recruited from the Linköping University Campus student pool. Inclusion criteria were right-handedness, normal hearing, native Swedish language, no serious tinnitus, no known neurological disease and no implants incompatible with MR-scanning. The study protocol was approved by the regional ethics review board (Dnr 2012/128). All participants gave written consent to take part in the study and all procedures followed standards set by the Declaration of Helsinki.

### Experimental Design

An experimental design with three conditions was used: one condition without visual task but with active listening to a sound sequence, one condition with a low demand visual task and one condition with a high demand visual task. The sound was also presented concurrently with the visual tasks, but here the participants were requested to ignore the sound. In addition, a silent/rest condition was used for baseline comparisons.

### Materials and Tasks

#### Sound

The background sound played back to the participants comprised a rapidly presented sequence of tones (for an easy way to get an idea of what the tone sequence sounded like to the participants, read “DRRRRRRRRRRRRR…” aloud). Two tone bursts, presented binaurally, were used for stimulation, a 1.0 kHz stimulus (standard tone) and a 1.2 kHz stimulus (deviant tone). The standard stimulus and the deviant stimulus consisted of four cycles of the tone: one cycle rise, two cycles plateau, and one cycle fall. The tone burst length was 4.0 ms for the 1.0 kHz stimulus and 3.33 ms for the 1.2 kHz stimulus. The 1.0 kHz tone was presented rapidly and repeatedly, with a presentation rate of 39.9 stimulus/s. However, once every 2–9 s the standard tone was replaced by the deviant tone. During these time windows, the deviant tone was presented repeatedly and rapidly, with a presentation rate of 39.9 stimulus/s, for a total of 1002.5 ms. Thereafter, the standard tone was again presented until the next deviant tone block occurred. The purpose of the deviant tone blocks was to control that the participants properly followed the task instructions when performing the active listening task.

#### Active Listening Task

In the active listening condition, participants were instructed to focus on the sound sequence and to rest their eyes on a fixation cross on the screen. To make sure that the participants were indeed listening to the sound, they were requested to indicate with their right hand’s index finger whenever the frequency of the tones changed (i.e., increased from 1 to 1.2 kHz).

#### Visual Working Memory Task

In the visual working memory task, a sequence of letters (drawn pseudo-randomly from the set: k, m, q, r, s, t, w) were presented visually. Each letter was presented individually. Participants were requested to press yes (using right hand’s index finger) or no (using right hand’s middle finger) in response to each presented letter, to indicate whether the presented letter was a target (i.e., the same letter as the letter* n* steps back in the sequence) or not a target (i.e., a letter that did not meet the criterion). In the low demand version of the task, *n* was 1. In the high demand version, *n* was 3. The visual sequences also contained “lures” (i.e., items that would meet the criterion for being a target in the other of the two task difficulty conditions but not in the present task difficulty condition). The presentation order of the letters was counterbalanced over the experiment and their presentation was organized into nine 8-item-lists (each including 2 targets and 0 lures), 18 12-item-lists (each including 3 targets and 1 lure), and 15 16-item lists (each including 4 targets and 2 lures). The total number of lists was 42 (528 trials in total), half was presented in the 1-back condition and half in the 3-back condition.

#### Task During Silence/Rest

In the silent/rest condition, no sound sequences were presented. The participants were asked to rest, with their eyes fixating a cross in the center of screen.

### Magnetic Resonance Imaging (MRI) Scanner Data Collection and Analysis

#### Procedure

A sparse imaging design (Hall et al., 1999) was used, to exclude measurement of scanner noise, where the activation measured derives from stimuli presented in a silent period between successive scans. The length of the stimuli presentation, the inter-stimulus intervals and the number of stimuli for each TR was setup to fit with time to scan one volume (2352 ms): presentation of each stimulus for 2000 ms, a 352 ms inter-stimulus interval, and four such events for each volume (*TR* = 9408 ms). The order of the conditions followed a pseudo-randomized sequence where 1-back, 3-back and active listening were each followed by each of the other conditions six times, and where silent/rest preceded the other conditions eight or nine times and followed the other conditions eight or nine times (Table 1). The duration of 1-back, 3-back and active listening differed between two, three or four TR (Table 2), and the durations were distributed in such a manner that a condition of a specific duration was never followed by a condition of same duration (Table 3).

**Table 1:** **Balancing the occurrences of conditions (making the probability for following conditions equal in relation to the frequency of each condition)**.

	Condition i + 1			
Condition i	Active listening	1-back	3-back	Silent/rest
Active listening		6*	6	9
1-back	6		6	9
3-back	6	6		8
Silence/rest	9	8	9	

*Condition 1 is followed by condition 2 six times.

**Table 2:** **The durations of each condition**.

	Durations			
Condition	1 TR	2 TR	3 TR	4 TR
Active listening		6	9	6
1-back		6	9	6
3-back		3	9	9
Silence/rest*	11	10		

*Silence/rest is included seven times in each run. At the first run 4 of those are 2 TR and at the second and the third run 3 of those are 2 TR. The remaining ones are 1 TR.

**Table 3:** **Distribution of durations following each other in the design given in TR**.

	Duration i + 1			
Duration i	1	2	3	4	Total
1	−	5	3	3	11
2	3	−	8	9	20
3	5	9	−	8	22
4	3	8	6	−	17
Total	11	22	17	20	

#### Stimulus Presentation, Data Collection and Analysis

The MR imaging was performed on a Philips Ingenia 3.0 Tesla scanner with a standard eight element head coil at the Centre for Medical Image Science and Visualization (CMIV) at Linköping University, Sweden. T2*-weighted functional images were acquired using a Gradient echo EPI sequence, with in-plane resolution of 3.0 × 3.0 mm; slice thickness of 3.0 mm with enough slices (40) to cover the whole brain; echo time (TE) = 40 ms; number of image volumes per session = 237. The slices were horizontal oblique. In addition to the functional data, a whole-brain 3D T1-weighted anatomical image (voxel sized of 1 × 1 × 1 mm^3^, *TR* = 25 ms, *TE* = 4.6 ms, 175 sagittal slices) was acquired for each participant at the start of the session.

All visual stimuli were projected to the participants on a screen which participants viewed via a mirror. Auditory stimuli were presented via NordicNeuroLab headphones (calibrated to 65 dB at 1 kHz). The presentation of the visual as well as the auditory stimuli was controlled using E-prime 2.0 software (Psychology Software Tools, Pittsburgh, PA, USA).

The statistical analysis comprised two steps. First, an overall, whole brain analysis was conducted. This analysis was supplemented by a Regions of Interest (ROI) analysis, involving brain areas of particular interest in the present context. The whole brain analysis employed FWE correction, ROI analyses employed Bonferroni correction of omnibus analysis of variance (ANOVA) results. Hence, for the three ROI conducted, a *p*-value smaller than 0.01 was considered statistically significant.

All image analyses were performed using SPM8 software (Wellcome Department of Imaging Neuroscience, University College, London, UK). Images were realigned, coregistered, normalized and smoothed (10 mm FWHM Gaussian kernel) following SPM8 standard pre-processing procedures. The analyses were conducted by fitting a general linear model (GLM) with regressors representing each of the four conditions, six movement parameters (derived from the realignment procedure) were included to control for movement during the session, and three regressors were used to remove the mean signal from the three runs. A high-pass filter with a cut-off of 128 s was modeled to eliminate low-frequency signal confounds such as scanner drift. These models were then fitted using a least-mean-squares method to each individual’s data, and parameter estimates were obtained.

#### Whole Brain Analysis

Contrasts for each experimental condition ([Condition > Baseline]) were defined individually for each participant. The individual contrast estimates from the first level analysis were then entered into a second level analysis involving the following contrasts: Active listening > silence; 3-back > 1-back; 3-back > silence; 1-back > silence; active listening < silence; 3-back < 1-back; 3-back < silence; and 1-back < silence. These four contrasts were analyzed by means of four one-sample *t*-tests.

#### ROI Analysis

For the first four contrasts, ROI beta values were extracted by means of the Marsbar software (Brett et al., 2002). Individual ROIs were extracted with the SPM Anatomy Toolbox 2.0 probability atlas (Eickhoff et al., 2005; see this citation for references to the individual areas). Auditory areas were: TE1.0, TE 1.1, TE 1.2, and TI1. In addition, ROIs were extracted for the Amygdala, and, for reference, BA2. BA2, which is located in the lateral postcentral gyrus and is a prominent part of the somatosensory cortex, was used as a reference, since we did not expect to find effects of sound or effort in this sensory region. Statistical analysis of contrast-related beta values were undertaken by means of two-way or one-way within groups ANOVA, using Greenhouse-Geisser correction of degrees of freedom where the sphericity assumption was violated.

## Results

### Whole Brain Analysis

The results, corrected for familywise error rate (FWR) from the four comparisons of primary interest for the purposes of this study (active listening > silence; 3-back > 1-back; 3-back > silence; 1-back > silence), are presented in Table 4. The contrasts are based on the second level whole-brain analysis.

**Table 4:** **Whole brain analysis of the effects of active listening and cognitive load in the *n*-back working memory task, FWE corrected values at *p* < 0.05 (*T* = 5.3)**.

Brain region	Cluster size	*Z*	*p*-value	*X*	*Y*	*Z*
**Sound (Active listening > Silence)**
L insula	2353	7.7	0.001	−32	22	1
L superior temporal gyrus				−59	−38	16
L transverse temporal (Heschl) gyrus				−35	−32	13
R superior temporal gyrus	2979	7.39	0.001	67	−29	13
R insula				37	22	1
R anterior insula				40	10	1
R medial frontal gyrus (BA 8)	894	6.67	0.001	4	31	43
R superior frontal gyrus (BA 6)				4	10	58
R superior frontal gyrus white matter				7	19	49
**Working memory (3-back > 1-back)**
Cingulate gyrus	2847	7.37	0.001	1	22	46
L anterior insula				−32	22	−2
R middle frontal gyrus				31	7	52
Inferior parietal lobe (BA 7)	1573	9.86	0.001	34	−62	43
Precuneus				7	−65	52
Inferior parietal lobe (BA 40)						
R thalamus	40	5.21	0.014	10	−23	16
L anterioventral thalamus	27	5.17	0.036	−8	−17	16
**3-back > silence**
L anterior insula	2960	7.59	0.001	−32	22	1
R anterior cingulate cortex				4	19	46
R middle frontal gyrus				31	7	58
L supramarginal gyrus	618	7.12	0.001	−50	−47	52
L Inferior parietal lobe (BA 7)				−32	−62	46
L Inferior parietal lobe (white matter)				−32	−44	40
L Inferior parietal lobe (white matter)	535	7.00	0.001	37	−53	43
R Inferior parietal lobe (white matter)				49	−44	46
R cerebellum (declive)	40	6.1	0.01	37	−62	−26
R lentiform	29	5.68	0.03	19	4	16
L cerebellum (declive)	26	5.62	0.04	−35	−62	−29
L middle frontal gyrus	77	5.13	0.001	−41	52	1
L middle frontal gyrus (BA10)				−32	49	10
**1-back > silence**
Left supplemental motor area (BA 6)	244	6.16	0.001	−8	13	46
Left supplemental motor area				−5	7	52
L anterior insula	163	5.79	0.001	−29	19	10
L insula (BA 13)				−38	13	7
L insula (BA 13)				−44	4	4
L inferior occipital lobe	116	5.71	0.001	−29	−89	−8
L inferior occipital gyrus				−41	−74	−8
L fusiform gyrus				−41	−53	−17
L rolandic operculum	42	5.51	0.003	−32	−32	16
L postcentral gyrus	125	5.26	0.001	−47	−32	52
L inferior parietal white matter				−32	−44	40
L inferior frontal gyrus (BA 9)	70	5.24	0.001	−56	7	34
R cerebellum (culmen)	50	5.16	0.002	22	−53	−29
R cerebellum (culmen)				34	−56	−29
R transverse temporal gyrus	29	5.04	0.011			

*Note. p-value denotes familywise error rate (FWR) corrected probabilities. L and R indicate left and right hemisphere, respectively. BA denotes Brodmann area; X, Y, and Z coordinates are given in MNI space*.

The difference between the active listening and the silent condition reflects the effects of sound. The difference between the 3-back and the 1-back condition is the number of items that have to be maintained in working memory to meet the task requirements. The visual and auditory input is the same, but cognitive load differs between the two conditions. Hence, this contrast represents the effects of working memory. The remaining contrasts—3-back vs. silence and 1-back vs. silence—were used to construct the working memory contrast, superimposed on sound.

**Figure 1:**
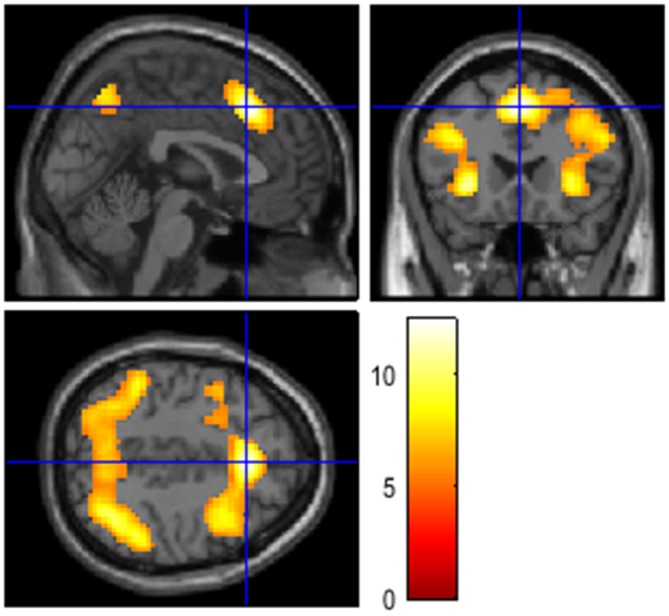
**The figures show involvement of cortical areas typically related to activation in working memory and attention-demanding tasks, including prefrontal and parietal cortices.** The contrast resulted from a one-sample *t*-test, testing for the effect of working memory load (3-back > 1-back, *T* > 5.31, FWE-corrected *p* < 0.05, *k* = 0, coordinates see Table 4).

The difference in neural activation between these two conditions aligned with this assumption. Activation in a frontoparietal network, typically associated with working memory involvement, was highest in the high cognitive load condition (3-back) in comparison with the low cognitive load condition (1-back). The 3-back > 1-back contrast resulted in activation of a network typically involved in effortful attention tasks, spanning the anterior insula, the anterior cingulate, the inferior parietal cortex, and the anterior thalamus (Figure 1). The remaining 3-back and 1-back contrasts represent combinations of these results. Finally, active listening produced activation across a wide network of areas in the temporal, insular, and frontal cortices. The behavioral data for the *n*-back task was lost due to technical errors. Therefore, behavioral data cannot be used as a manipulation check, but the comparisons reported in Table 4 confirm the success of the cognitive load manipulation.

### ROI Analysis: Effects of Cognitive Load on Distractor Processing

**Figure 2:**
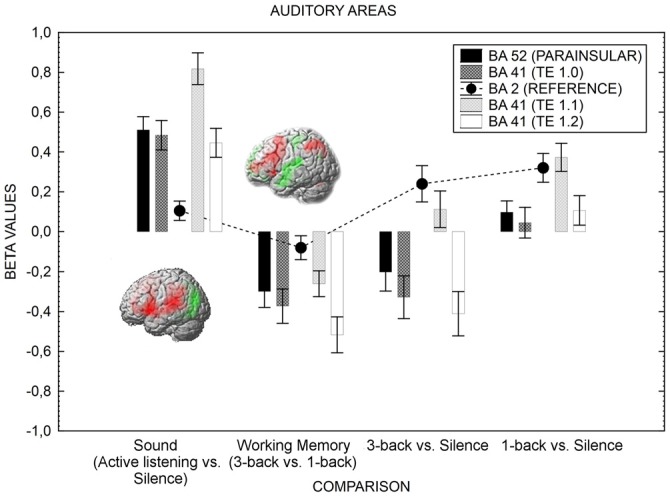
**The figure shows the auditory cortex’s activation in various conditions.** The activity is greater when attention is directed toward the sound (active listening) in comparison with when it is directed away from the sound toward a visual task (1-back). When the difficulty of the visual task increases (3-back), the neural activity of the auditory cortex is further suppressed. As the *n*-back task involves sub-vocal rehearsal, and to preserve statistical power, the left hemisphere only is shown, but the result pattern was the same for right hemisphere. Error bars represent standard error of means. Activation (red) and deactivation (green) is rendered on the left hemisphere for the active listening vs. silence contrast and the 3-back vs. 1-back contrast. The contrasts displayed in the Figure resulted from four one-sample *t*-tests, testing for the effect of sound (active listening vs. silence, *T* = 5.39, FWE corrected *p* < 0.05, *k* = 0), working memory load (3-back > 1-back, *T* = 5.31, FWE corrected *p* < 0.05, *k* = 0), 3-back vs. silence (*T* = 5.30, FWE corrected *p* < 0.05, *k* = 0), and 1-back vs. silence (*T* = 5.40, FWE corrected *p* < 0.05, *k* = 0). The coordinates for the contrasts are shown in Table 4.

**Figure 3:**
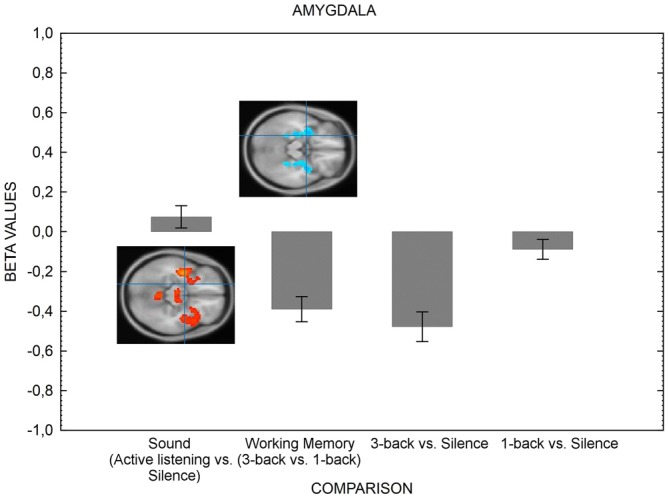
**The figure shows the amygdala’s activation in various conditions.** The left hemisphere only is shown for simplicity, but the result pattern was the same for right hemisphere. The suppression of amygdala is greater when the cognitive load of the visual-verbal working memory task is high in comparison with when load is low. Error bars represent standard error of means. The inserts shows results (bars indicate means of parameter estimates (±SEM, arbitrary units) at the level of the amygdala (crossmark). Activation is depicted in red and deactivation is depicted in blue for the active listening vs. silence contrast and the 3-back vs. 1-back contrast. As can be noted, there is no discernable activation in the active listening vs. silence contrast. The contrasts displayed in the Figure resulted from four one-sample *t*-tests, testing for the effect of sound (active listening vs. silence, *T* = 5.39, FWE corrected *p* < 0.05, *k* = 0), working memory load (3-back > 1-back, *T* = 5.31, FWE corrected *p* < 0.05, *k* = 0), 3-back vs. silence (*T* = 5.30, FWE corrected *p* < 0.05, *k* = 0) and 1-back vs. silence (*T* = 5.40, FWE corrected *p* < 0.05, *k* = 0). The coordinates for the contrasts are shown in Table 4.

We now turn to the test of the main hypothesis of the current study; higher visual-verbal cognitive load should suppress activation in areas serving auditory processing. Figure 2 presents the results for the ROIs related to the auditory areas and Figure 3 depicts the amygdala ROI.

As can be seen in Figure 2, the neural activity of the primary auditory cortex was more suppressed when the task-difficulty of the visual task was high (3-back) in comparison with when the task-difficulty was low, even though the sensory input is the same in the two task difficulty conditions. As a control, Figure 2 also displays the difference between active listening (no *n*-back task) and 1-back, and suggests that the auditory cortex was more responsive to the sound when the locus-of-attention was set on the sound (active listening) in comparison with when it was set on visual stimuli (1-back). A repeated measures 4 × 4 (Comparison by Auditory Areas) analysis of variance disclosed a two-way interaction, *F*_(3.81,118.32)_ = 5.44, *p* < 0.01, in addition to statistically significant main effects: Comparison *F*_(1.97,61.09)_ = 50.72 *p* < 0.01; Auditory Areas *F*_(2.45,75.89)_ = 24.04, *p* < 0.01. Of particular importance to the purpose of this study, Scheffé *post hoc* test disclosed statistically significant differences between all auditory areas when the sound (active listening vs. silence) contrast was compared to the working memory (3-back vs. 1-back) contrast (all *p*’s < 0.01). The 3-back vs. sound contrast mimicked the working memory contrast whereas the 1-back vs. sound depicted an attenuated sound contrast.

Figure 2 also displays, for reference purposes, how the primary somatosensory area (BA 2) does not respond to the experimental manipulations the same way as the primary auditory cortex does (this region, BA2, was not included in the ANOVA). For BA2, the effect of Contrast was statistically significant: *F*_(2.19,67.93)_ = 11.02, *p* < 0.01. Scheffé *post hoc* comparisons revealed that the 3-back vs. Silence and 1-back vs. Silence comparisons were different from the Active listening vs. Silence comparison and the 3-back vs. 1-back comparisons (*p* < 0.05). While active listening and cognitive load failed to produce signal changes in BA2, the added cognitive load of sensory feedback related to motor responding yielded a slight activation in BA2. Hence, the effects of the cognitive load manipulation are selective.

The hypothesis that concentration-requirements also suppress the activity in amygdala was also confirmed (Figure 3). The activity in amygdala was suppressed when the difficulty of the visual-verbal working memory task was high (3-back) in comparison with when it was low (1-back), as confirmed by an ANOVA for the difference between the four comparisons of interest, *F*_(1.97,61.16)_ = 24.38, *p* < 0.01. Taken together, the results suggest that the cognitive system protects goal-directed behavior by shielding itself from both exogenous and endogenous sources of distraction.

## Discussion

The results show that the selective attention toward an auditory stimulus facilitates neural processing in the auditory cortex, consistent with the assumption that the neural activity in areas serving target processing is “boosted” when the target is selectively attended (Gazzaley et al., 2005). When attention is instead localized to a visual input, the neural response in the auditory cortex is lower than when the auditory input is actively attended. This is consistent with the idea of a cross-modal suppression of the neural activity responding to the distractor modality (Weissman et al., 2004). Moreover, when cognitive load of the visual-verbal working memory task is higher, the neural response to the auditory stimulus is further suppressed, even though the visual input is the same as when cognitive load is lower. Finally, the results also suggest that amygdala activity decreases when cognitive load escalates. Taken together, the results suggest that higher cognitive load shields against distraction, as a side-effect of the recruitment of a network typically associated with effortful attention and working memory processing (middle frontal gyrus, the anterior insula, the cingulate, and the inferior parietal cortex).

The results reported here expand on a previous study on the effects of cognitive load on brainstem activity (Sörqvist et al., 2012) by showing that higher cognitive load in a visually-presented task suppresses task-irrelevant processing at the cortical level (auditory cortex) as well as at the sub-cortical level (amygdala). As such, the results speak to the debate about the effects of cognitive load on distractor processing. One group of researchers argues that cognitive load increases undesired responsiveness to distractors in the periphery (e.g., Lavie et al., 2004; Lavie and De Fockert, 2005; Dalton et al., 2009; Sabri et al., 2014); while another group of researchers argues that cognitive load decreases distractor processing (e.g., Kim et al., 2005; SanMiguel et al., 2008; Sörqvist and Marsh, 2015). Although the current experiment cannot resolve these inconsistencies, the experiment reported here supports the view that cognitive load decreases distractibility.

One possibility is that cognitive load has different effects depending on whether the to-be-attended and the to-be-ignored stimuli are presented in the same modality or in different modalities. Evidence in favor of the view that cognitive load decreases distractor processing, comes from studies wherein target and distractor information are presented in different modalities (the current study, and extant research; Kim et al., 2005; SanMiguel et al., 2008; Regenbogen et al., 2012; Sörqvist et al., 2012; Halin et al., 2015). When target and distractors are presented in different modalities, the stimulus-competition is low (i.e., the cognitive system can easily distinguish target from non-target stimuli). This could facilitate the suppression of distractor processing, because the distractor modality is clearly distinguishable from the target modality (cf., Schwartz et al., 2005).

Conversely, evidence in favor of the view that cognitive load increases distractor processing comes from studies wherein target and distractor information are presented in the same modality. In this case, higher cognitive load increases the neural responses toward the distractors (Sabri et al., 2014). The reason for this could be that the system is insufficiently fine-tuned to hit the distractor processing neurons with suppression (cf., Parks et al., 2013).

It should also be noted that the timing between distractor presentation and target presentation modulates the magnitude of distraction. For example, when a sequence of words is presented visually, and the participants’ task is to recall the words, recall is more impaired by auditory distractor-words when the distractor words are presented simultaneously with the visual words, in comparison with when the distractor words are sandwiched in-between the visual words (Marsh et al., 2015). In the current study, the visual task and the auditory distractors were presented simultaneously. It is unclear whether cognitive load would have the same effect on distractor processing if the presentation of the task-materials and the presentation of the distractor materials are chronologically separated.

The similarity between the targets and the distractors can also play a role (Kim et al., 2005; Park et al., 2007). In the current study, the items in working memory (letters) and the distractors (tone sequences) were unrelated, and did, arguably, not share features. It is unclear whether cognitive load would have had a different effect if the items in working memory and the distractors would have shared features.

There is yet another crucial difference between the studies in favor of the view that cognitive load increases distraction and those in favor of the view that cognitive load decreases distraction. Studies showing that cognitive load decreases distraction, have studied the effects of cognitive load when the participants are performing a single task. Here, distractor processing is not evaluated by its effect on a secondary task. Instead, distractor processing has been measured by task-unrelated neural responses (the current study; SanMiguel et al., 2008; Sörqvist et al., 2012), by long-term memory of what has been presented in the to-be-ignored channel (Halin et al., 2015), or by the behavioral effects of distractors on performance in the task that is used to manipulate cognitive load (Kim et al., 2005).

Studies suggesting that cognitive load increases distractibility have instead often used a dual-task setting (Lavie and De Fockert, 2005; Dalton et al., 2009). Here, the participants perform one task that is used to manipulate cognitive load, while they also perform a concurrent task that is used to measure distraction. For example, a working memory task can be used to manipulate cognitive load by requesting the participants to maintain either a small set or a large set of items in memory, and a visual search task is performed during the working memory retention interval. Distraction is operationalized as the effect visual distractors have on response times in the visual search task, not as the cost the distractors cause to the working memory task.

Attentional engagement could be responsible for this difference between single-task and dual-task studies. In a dual-task study, executive resources must be spent on two sources of task-relevant information. Therefore, the attentional engagement is low in the task that is used to measure distractibility, especially when the cognitive load is high in the task which is not used to measure distractibility. And a consequence of this low attentional engagement is greater susceptibility to distraction (Sörqvist and Marsh, 2015). Conversely, in a single task study, the executive resources can be fully focused upon a single source of task-relevant information. Consequently, a high state of focal-task engagement can be reached. And a consequence of this high attentional engagement is lower susceptibility to distraction (Sörqvist and Marsh, 2015), as seen in a suppression of the neural processing of distractors (current study; SanMiguel et al., 2008 ; Sörqvist et al., 2012) and other indices (Kim et al., 2005; Halin et al., 2015).

The effects of the upward shift in concentration—in response to a rise in task difficulty—are not limited to cortical areas of the brain. They also expand to suppression of subcortical areas such as the amygdala (Kellermann et al., 2012) and the brainstem (Sörqvist et al., 2012). The amygdala is suppressed when the cognitive system is challenged, perhaps because the system inhibits emotional responses to protect the current goal-directed behavior (Van Dillen et al., 2009; Okon-Singer et al., 2013). The study reported here provides further evidence for the view that higher cognitive load suppresses amygdala activity, and the study shows that cognitive load does so even when the distractor stimulus is non-emotional.

In conclusion, higher demands for concentration appear to recruit a dynamic fronto-insular neural network and—perhaps as a side effect—suppress peripheral processing at cortical and subcortical areas. Therefore, higher cognitive load shields against distraction, at least distraction to a task that can be fully attended to. The ecological value of the suppression effects of cognitive load would be to protect the cognitive system when it is challenged by a demanding task, so that a desired goal can be reached. This protection would arguably require not only an act on current distractors (e.g., a present background sound) but also potential future distractors (e.g., the possibility of emotionally alarming stimuli), which could be why the suppression acts on both cortical and subcortical levels.

## Author Contributions

PS and JR conceived the study. ÖD and TK designed and performed the experiment. ÖD and TK analyzed the results. PS, ÖD, TK, and JR wrote the manuscript.

## Conflict of Interest Statement

The authors declare that the research was conducted in the absence of any commercial or financial relationships that could be construed as a potential conflict of interest.
